# Dermatological Implications of the Taqiyah and Imamah: Recommendations for Delivering Culturally Conscious Care

**DOI:** 10.7759/cureus.45528

**Published:** 2023-09-19

**Authors:** Bilal Irfan, Aneela Yaqoob

**Affiliations:** 1 Microbiology and Immunology, University of Michigan, Ann Arbor, USA; 2 Infectious Diseases, Beaumont Hospital, Taylor, USA

**Keywords:** religious support, culturally adaptive, culturally competent care, imamah, taqiyah

## Abstract

Background and objective

The growing cultural and religious diversity in healthcare settings necessitates clinicians to integrate cultural competence and sensitivity into their practice. Despite significant research focusing on the hijab worn by Muslim women, there is a gap in understanding the dermatological implications of the taqiyah and imamah, worn by Muslim men. In light of this, this study aimed to offer insights into delivering culturally conscious dermatological care for Muslim men wearing these garments, thereby bridging a crucial knowledge gap.

Materials and methods

The study employed a comprehensive research strategy that encompassed both medical literature and foundational Islamic texts. PubMed, Web of Science, and Scopus were used for medical literature searches, while al-Maktabah al-Shamela and Sunnah.com, along with Quranic and Hadith translations were consulted for religious insights. A thematic analysis was employed to identify patterns, challenges, and unique points, ensuring a holistic understanding of the subject.

Results

Our findings revealed that wearing a taqiyah or imamah has both beneficial and detrimental dermatological effects, depending on factors such as climate, fabric, and hygiene practices. While the garments are rooted in Islamic tradition, their use varies based on cultural context rather than strict religious guidelines. Moreover, gender dynamics and the concept of privacy ('awrah) within Islamic teachings have implications for healthcare interactions. The study offers practical guidelines for dermatological care tailored to Muslim men wearing a taqiyah or imamah. It emphasizes the importance of material choice, hygiene practices, and the willingness of many Muslim men to be accommodating in medical settings, albeit with some reservations. The paper also discusses the role of telemedicine in culturally sensitive healthcare delivery, recommending measures such as secure communication channels and self-imaging options.

Conclusion

The paper provides comprehensive recommendations aimed at delivering culturally and religiously sensitive dermatological care to Muslim men wearing a taqiyah or imamah. By integrating both medical best practices and a nuanced understanding of Islamic customs, healthcare providers can foster a more trusting and effective care relationship, thereby improving patient satisfaction and dermatological outcomes.

## Introduction

In an increasingly interconnected world, healthcare practitioners often encounter the challenge of offering medical care to a multifaceted tapestry of individuals with diverse cultures, customs, and religious practices. This complexity has foregrounded the necessity for clinicians to move beyond mere technical proficiency and delve into the realms of cultural competence and sensitivity. Cultural nuances, particularly religious beliefs, can heavily influence an individual's approach to health, well-being, and their interaction with the healthcare system. Such interplay between religion and healthcare is very evident in Muslim communities, which constitute approximately a quarter of the world's population [1].

The importance of cultural and religious sensitivity in healthcare cannot be overstated [2]. A clinician's understanding and respect for religious customs not only ensure that the provided care is holistic but also fosters a relationship of trust and mutual respect with the patient [3]. In the context of dermatological care, understanding the religious significance and practices associated with the taqiyah and imamah, worn by many Muslim men, is paramount. These religious garments, while serving as a marker of religious identity, can also play a role in the dermatological health of wearers, impacting the conditions of the scalp and skin underneath [4]. The taqiyah, a rounded skullcap, and the imamah, a turban-like cloth, are not just mere adornments but have deeply ingrained significance in Islamic culture, reflecting the wearer's faith, devotion, and sometimes regional identity.

While there is substantial focus on the hijab, primarily worn by Muslim women, and its implications in healthcare, the nuances associated with the taqiyah and imamah remain less explored in medical literature [5]. This gap poses challenges in delivering optimal care to Muslim male patients, whose needs and preferences might differ based on their religious adherence, cultural background, and personal beliefs. This paper aims to bridge this knowledge gap, offering insights into how healthcare professionals can tailor their approach to ensure culture-aware dermatological care for Muslim men wearing the taqiyah or imamah. By exploring the intricacies of these religious headgears, the study hopes to underscore the importance of personalizing medical care in accordance with the cultural and religious sensitivities of the patient.

## Materials and methods

The intersection of dermatology and religious practices, particularly in the context of the Islamic garments taqiyah and imamah, demands a methodical exploration of both medical literature and foundational Islamic texts. To that end, our study embarked on a twofold mission: examining the implications of wearing these head coverings in dermatological contexts and probing their religious underpinnings.

We relied on an array of digital libraries including PubMed, Web of Science, and Scopus to gather medical studies or papers that touch on the dermatological ramifications or considerations for wearing head garments. Religious nuances in terms of guidelines and customs for wearing an imamah were uncovered by parsing through Hadith collections, the documented sayings and practices of the Prophet Muhammad (peace be upon him). The Quran, alongside Tafsirs (exegesis) highlighting interpretations of the scripture were particularly significant in helping to form an understanding of the intricacies of modesty requirements in Islamic tradition. Sunnah.com and Quran.com served as useful tools for searching keywords and references to male head coverings, particularly for canonical texts such as Sahih al-Bukhari and Sahih Muslim. Fatwas (Islamic legal edicts) issued by prominent Muftis or jurists, forming the ulema (scholarly body), detailing laws and practical implementation were particularly relevant in ensuring that the recommendations were fully in accordance with the tenets of the Islamic faith.

Our search enabled us to synthesize the information gathered and conduct a thematic analysis by organizing data from the medical literature and Islamic texts. Through identifying patterns, overlaps, and points of distinction, we were able to sustain a holistic understanding while weaving together medical implications with religious tenets and practices. Nevertheless, we certainly faced certain challenges as reliance on translated Islamic texts may mean certain nuances could be overlooked or misconstrued. Hence, we excluded texts that were not directly relevant to the topic at hand or those not available in Arabic, Urdu, or English. Islamic jurisprudence encompasses a vast body of thought with different schools that offer varied perspectives on issues relating to medical practice, and thus while endeavoring for a comprehensive understanding, certain nuances or perspectives might not be as extensively represented as others.

## Results

An analysis of the Quranic and Hadith literature, coupled with Islamic legal texts and medical best practices aided in providing a comprehensive understanding of the dynamics between dermatology and the use of religious headgear. In applying this to the taqiyah and imamah, our findings can be categorized into two pivotal segments: potential dermatological ramifications for users and insights for care practices drawn from religious and cultural contexts.

The continual use of a turban-like headgear has shown varied outcomes concerning scalp health [6]. In certain contexts, these head coverings appear beneficial, aiding in the retention of scalp moisture or the prominence of Staphylococcus capitis, which consequently could aid in producing antimicrobial peptides [7,8,9]. However, when worn in humid climates or during strenuous activities, the reduced ventilation can sometimes aggravate existing conditions such as seborrheic eczema, tinea capitis, or traction alopecia, largely attributed to increased sweat and sebum production [10]. An essential component of maintaining good health is the criticality of hygiene. The frequent washing of these head coverings, be it weekly or after every few uses could reduce the risk of scalp infection [11]. Delving further into the material aspect, it becomes evident that the fabric of the taqiyah or imamah plays a crucial role in skin outcomes. Natural fabrics like cotton and linen, preferred for their breathability, are associated with optimal scalp health, whereas certain synthetic or densely woven materials sometimes foster conditions conducive to skin issues due to excessive sweating [12].

Direct references to male head coverings like the taqiyah and imamah are limited in the Quran and Hadith, despite the scriptures emphasizing overarching themes of modesty and personal comportment [13]. Our analysis suggests that while these coverings are deeply rooted in Muslim traditions and seen as emblems of piety, humility, and identity, their adoption can vary based on cultural customs rather than strict religious injunctions. The diverse Islamic jurisprudential discourses reflect a consensus on the recommended status of the taqiyah, especially during prayers, without it being a strict mandate. The imamah, traditionally donned by scholars or in specific cultural contexts, embodies both religious symbolism and societal roles. This inherent flexibility implies that many adherents might be accommodating during medical procedures, with some reservations rooted in religious teachings. A prominent theme in the literature is the gender dynamics in healthcare interactions. Many individuals displayed a proclivity for same-gender healthcare providers, a preference deeply anchored in Islamic guidelines emphasizing modesty and minimal physical interactions between unrelated members of the opposite gender [14]. Furthermore, cultural distinctions surface, with variances in the significance and practices surrounding these head coverings across Muslim-majority regions. For instance, the taqiyah's ubiquity is evident in South Asian and Middle Eastern cultures, while the imamah holds more sway in certain Gulf and North African regions.

Both the textual sources and patient accounts consistently underlined the importance of privacy and comfort in medical settings. The Islamic notion of 'awrah, denoting the intimate regions of an individual, spanning from the navel to the knees for men, is also emblematic of a broader ethos of personal dignity and space [15]. This respect for personal boundaries and individual privacy is a salient feature of Islamic teachings, and its implications are unmistakably profound in clinical interactions.

## Discussion

The delivery of dermatological care to Muslim men who wear a taqiyah or imamah offers a unique intersection of medical, cultural, and religious considerations, requiring a multidisciplinary approach. Our findings revealed certain practical guidelines and we propose recommendations for clinicians that can enrich healthcare provision by harmonizing evidence-based practice with respect for cultural and religious traditions. On the medical and dermatological front, our study underscores the necessity of tailoring care based on the specific needs and conditions of the scalp and skin. For instance, specific types of breathable, hypoallergenic fabric such as cotton or linen could mitigate adverse dermatological reactions, especially in patients with pre-existing conditions like dermatitis or tinea capitis. These suggestions should be included in educational materials offered during clinical visits to preemptively address potential issues [16]. Clinicians should also inquire about hygiene practices associated with these head coverings, emphasizing the importance of regular washing to minimize microbial infections (Table 1).

**Table 1:** Recommendations for delivering culturally conscious care to patients who wear a taqiyah or imamah in teledermatology and in-person clinical settings

Suggestions for clinicians
Offer pre-consultation appointments wherein patient concerns and questions regarding imaging or examination can be thoroughly addressed
Organize meetings, in-person or telehealth, in such a manner that limits non-provider visibility to the patients through carefully planned seating arrangements and tinted desktop screens
Bar transmissibility of conversation to the outside by closing doors and utilizing headphones in telehealth encounters as appropriate
Incorporate illustrations and visual aids that display Muslim men who wear a taqiyah or imamah to provide comfort and enhance accessibility
Be cognizant of language barriers that may hamper communication, and make efforts to include other family caregivers or language tools to ensure a collaborative process
Recognize that eye-to-eye communication and hand-shaking may not be customary and Muslim men may lower their gazes in opposite-sex interactions
Prioritize options for self-imaging and submitting wound pictures through secure platforms
Demonstrate respect for patient privacy by being cognizant of the male awrah (private parts) in Islamic tradition, encompassing the navel down to knees and inclusive of it
Encourage wearing imamahs made of comfortable cloth and wrapped less tight or with one or two extending knots that can be adjusted as needed for clinical encounters to avoid fully removing the piece
Empower patients with an option to write and detail the necessary scope of accommodations they might need before their appointment and their comfort levels with removing or adjusting a taqiyah
Maintain a single-stall restroom with a footbath that allows patients to change dress comfortably and conduct a ritual ablution (Wudhu), in addition to providing a room for prayer and spiritual services
Whenever possible, ensure access to male healthcare providers to provide patient comfort, privacy, and adherence to cultural preferences during medical consultations

Simultaneously, understanding the religious background related to the wearing of taqiyah and imamah is vital for clinicians. Our investigation into Islamic texts highlighted that these head coverings are recommended rather than obligatory, thus offering a degree of flexibility. Clinicians can leverage this understanding to engage in an open dialogue about the necessity of removing or adjusting these articles during medical examinations, always ensuring that the patient feels respected in their cultural and religious observances. Gender dynamics represent another layer of complexity, as some Muslim men may prefer not to be examined by healthcare providers of the opposite sex. While it may not always be possible to meet these preferences due to practical reasons, offering alternative methods like self-imaging options can build trust and increase adherence to treatment plans. Providers should also remain cognizant of non-verbal cues and customs that might be at play during such interactions.

Furthermore, as telemedicine increasingly becomes a relevant aspect of healthcare delivery, the principles of cultural sensitivity must extend beyond physical clinical settings [17]. Ensuring confidential communication channels and offering options for self-imaging prior to teleconsultations can go a long way in minimizing patient anxiety, particularly when it comes to respecting the Islamic concept of 'awrah, which specifies the areas of the body that should remain covered. In the physical healthcare environment, minor yet impactful changes can make a significant difference in patient comfort. Clinicians could consider implementing features like tinted desktop screens, secluded seating areas, and private rooms for ritual ablution (Wudhu), a component of spiritual practice in Islam. Such changes not only offer patients greater privacy but also align with their religious practices, thereby enhancing the overall patient experience.

## Conclusions

The need for delivering culturally sensitive healthcare is paramount in our increasingly diverse societies. This paper fills a significant gap in the existing medical literature by focusing on the dermatological care needs of Muslim men who wear a taqiyah or imamah. We offered practical recommendations that integrate dermatological best practices with a deep understanding of Islamic customs and values. These guidelines aim to promote a culturally responsive environment in both teledermatology and in-person settings, thereby improving patient satisfaction and overall dermatological outcomes. By recognizing and respecting the interplay of medical, cultural, and religious factors, healthcare providers can build a more trusting and holistic care relationship with their Muslim male patients.
